# Effect of miR-29b on the Proliferation and Apoptosis of Pulmonary Artery Smooth Muscle Cells by Targeting Mcl-1 and CCND2

**DOI:** 10.1155/2018/6051407

**Published:** 2018-01-31

**Authors:** Juan Chen, Yanping Li, Yun Li, Lijian Xie, Jianyi Wang, Yongwei Zhang, Tingting Xiao

**Affiliations:** Department of Cardiology, Shanghai Children's Hospital, Shanghai Jiao Tong University, 355 Lu Ding Road, Shanghai 200062, China

## Abstract

The proliferation and apoptosis of pulmonary artery smooth muscle cells (PASMCs) are considered to be key steps in the progression of pulmonary arterial hypertension (PAH). MicroRNAs (e.g., miR-29b) have been identified in various diseases to be critical modulators of cell growth and apoptosis by targeting Mcl-1 and CCND2. However, the role of miR-29b in PAH remains unknown. So we try to investigate the effect of miR-29b on Mcl-1 and CCND2 protein in PASMCs, analyze the effect of miR-29b on the proliferation of PASMCs, and explore the significance of miR-29b in the proliferation, apoptosis, and gene therapy of PAH. It was observed that gene chip analysis showed miR-29b expression in pulmonary artery tissue. The expression of miR-29b was significantly reduced in PAH model mice. MiR-29b inhibited the proliferation of PASMCs and promoted the apoptosis of PASMCs. Mechanically, miR-29b could inhibit the expression of Mcl-1 and CCND2 protein and silenced Mcl-1 and CCND2 could abolish the change of proliferation and apoptosis of PASMCs. These results demonstrate that miR-29b suppressed cellular proliferation and promoted apoptosis of PASMCs, possibly through the inhibition of Mcl-1 and CCND2. Therefore, miR-29b may serve as a useful therapeutic tool to treat PAH.

## 1. Introduction

Pulmonary arterial hypertension (PAH) is a devastating disease which leads to right ventricular hypertrophy, progressive heart failure, and potentially death [1]. Approximately 10%–15% of patients with PAH die within one year of medical follow-up, despite receiving treatment. The pathology of PAH is characterized by pulmonary vasoconstriction, in situ thrombosis, and vessel remodeling. In particular, vessel remodeling is associated with enhanced proliferation and reduced apoptosis of pulmonary artery smooth muscle cells (PASMCs) [2, 3].

Recently, microRNAs (miRNAs) have been identified as endogenous noncoding small RNAs, which contain 19–25 nucleotides and are involved in the posttranscription regulation of gene expression [4]. In addition, studies have confirmed that the deregulated expression of miRNAs may be involved in the pathogenesis of numerous cardiovascular diseases [5]. Indeed, the dysregulation of more than a dozen miRNAs has been reported in PAH lung specimens from both human and animal models [6]. Moreover, miR-1, miR-145, and miR-221/222 can play an important role in the proliferation and migration of smooth muscle cells [7–9].

MiR-29b has been found to be downregulated in a variety of diseases. Cushing et al. [10] demonstrated that the miR-29 family is suppressed by transforming growth factor- (TGF-) *β*1 in human fetal lung fibroblast IMR-90 cells and that many fibrosis-associated genes upregulated by TGF-*β*1 are depressed following a miR-29 knockdown. In addition, the expression of miR-29 in both gastric and rhabdomyosarcoma was also shown to be downregulated [11, 12]. Toyono et al. [13] found that the overexpression of miR-29b decreased extracellular matrix protein production in human corneal endothelial cells. Therefore, miR-29 replacement therapy may be a novel treatment strategy for Fuchs endothelial corneal dystrophy, aimed at reducing the pathological production of extracellular matrix proteins in the Descemet membrane. The overexpression of miR-29b reduces collagen biosynthesis by inhibiting heat shock protein 47 during wound healing in the skin [14]. CCND2 is a cell cycle regulator, and aberrant expression can lead to abnormal cellular proliferation. Researchers have found that the introduction of miR-29b in Ewing's Sarcoma (ES) cells could inhibit the c-Myc-mediated up-regulation of CCND2, thus preventing the progression of the cell cycle [15]. Mcl-1 belongs to the Bcl-2 family and is a key regulator of apoptosis. Previous studies have found that miR-29b can directly downregulate the translation of Mcl-1; this results in the inhibition of prostate cancer cells and tumor progression in an extrahepatic bile duct cancer cell line [16, 17].

In the present study, we hypothesized that miR-29b might participate in the vascular remodeling of PAH by targeting Mcl-1 and CCND2. An in silico analysis of the potential miR-29b targets (http://www.targetscan.org and http://www.microrna.org) revealed that both Mcl-1 and CCND2 are possible targets of miR-29b. However, since little is known regarding the mechanism of miR-29b in PAH, we investigated whether Mcl-1 and CCND2 are the direct targets of miR-29b. Moreover, we sought to elucidate whether miR-29b can influence the proliferation and apoptosis of PASMCs through targeting Mcl-1 and CCND2.

## 2. Materials and Methods

### 2.1. Animals

All animal husbandry and experimental procedures were performed according to protocols approved by the Animal Care and Use Committees Shanghai Children's Hospital. C57BL/6 male mice (eight weeks old) were purchased from the SLAC Laboratory Animal Company (Shanghai, People's Republic of China). The mice were fed with a standard chow diet and maintained on a 12 h light-dark cycle under controlled temperature condition.

### 2.2. Chronic Hypoxia-Induced PAH Mice Model

C57BL/6 male mice (eight weeks old) were exposed to 10% oxygen in a ventilated chamber. The chamber was opened twice a week for 10 min for cleaning and supple. Three weeks later, pulmonary hemodynamic changes and vascular remodeling were assessed. The control group was exposed to normal air. Moreover, miRNAs from the PA of the mice described above were isolated to analyze the expression of miR-29b.

### 2.3. MiRNA Microarray

With the use of a miRNA Isolation Kit (Omega, Stamford CT, USA), the total RNA was extracted from the pulmonary arteries of mice. The concentration of RNA was measured using a Nanodrop 2000 spectrophotometer (Thermo Scientific, Massachusetts, USA). Each microarray chip was hybridized with a single sample labeled with either Cy3 or Cy5. The microarray was performed by Usen Shanghai Biotech Company (Shanghai, China) using an Agilent human miRNA array (v.12.0).

### 2.4. Cell Culture and Treatments

Human PASMCs were purchased from ATCC (Manassas, VA, USA). The cells were directly plated on regular 60 mm cell culture dishes or 12-well plates kept under normoxia (21% O_2_) or hypoxic (1% O_2_) conditions in incubators that maintained a constant environment (5% CO_2_ balanced with N_2_) for the indicated periods of time. The cells were then transfected with miR-29b mimic, a miR-29b inhibitor, or the negative control (Biotend, Shanghai, China).

### 2.5. Quantitative Real-Time PCR

The miRNA was isolated from cells or tissues using a miRNA Isolation Kit. Taqman probes were used for measuring the mature miR29b and U6 snRNA expression. The reaction was performed using a miRNA assay kit (Riobio, Guangzhou, China) in accordance with the manufacturer's instructions. A SYBR Green PCR system (Applied Biosystems) involved a cycle at 95°C for 10 min, followed by 40 cycles at 95°C for 15 s, and a final cycle at 60°C for 60 s. The threshold cycle (Ct) of each target gene was automatically defined and located in the linear amplification phase of the PCR and then normalized to the U6 (DCt value) of each group. Mature miR29 expression was normalized to the U6 snRNA.

### 2.6. Cell Proliferation Assay

Cells of same density were seeded into 96-well plates and treated with either the miR29b mimic, inhibitor, or negative control at a final concentration of 100 nM for 48 and 72 h. For the cell proliferation assay, the cells were covered with 100 *μ*L fresh medium and 10 *μ*L of the Cell Counting Kit-8 (CCK-8) Assay Kit reagent (Dojindo, Tabaru, Japan) was added. The cells were then incubated at 37°C for 1 h and the absorbance was measured at 450 nm using a SpectraMax 190 Microplate Reader. The reference wavelength was 600 nm. Background absorbance (from wells without cells) was subtracted from all values.

### 2.7. Apoptosis, Cell Cycle Analysis

For the analysis of apoptosis, the cells were resuspended in Binding Buffer (Apoptosis Detection Kit; BD Pharmingen, San Diego, USA) at a concentration of 10^6^ cells/mL and 5 *μ*L of FITC AnnexinV and 5 *μ*L Propidium Iodide were added to 100 *μ*L of the cell suspension. A volume of 400 *μ*L Binding Buffer was added after a 10 min incubation in the dark. Apoptosis was analyzed by flow cytometry (Becton-Dickinson, NJ, USA) using Cell Quest software. For cell cycle analysis, the cells were fixed in 70% ethanol and stored at −20°C for 1 h. The fixed cells were washed with PBS, treated with RNase A (50 *μ*g/mL), and stained with PI (50 *μ*g/mL) for 30 min in the dark. The stained cells were analyzed by flow cytometry.

### 2.8. Western Blotting

Proteins from the total cell lysates were separated by 10% SDS-PAGE and probed with different primary antibodies against MCL-1, CCND2, and GAPDH (Cell Signaling Technology, MA, USA).

### 2.9. Luciferase Assay

PASMCs were seeded into 24-well plates. A pGL3-luciferase plasmid (Biotend) and* Renilla* luciferase control plasmid were cotransfected into the cells using a Lipofectamine 2000 (Invitrogen, Waltham, MA) with the miR-29b mimic, inhibitor, or negative control as indicated. After incubation for 24 h, the luciferase activity was measured by performing a dual-luciferase assay using a Glomax96 Microplate Luminometer (Promega). Firefly luciferase activity was normalized to the* Renilla* luciferase activity.

### 2.10. Statistical Analysis

The data are expressed as the mean ± SEM. The time course was analyzed by a two-way ANOVA, and a Student's *t*-test was used for all other statistical analyses (GraphPad Prism 5.0 software). A value of *p* < 0.05 was considered to be statistically significant.

## 3. Results

### 3.1. MiR-29b Is Downregulated in PAH

**Figure 1:**
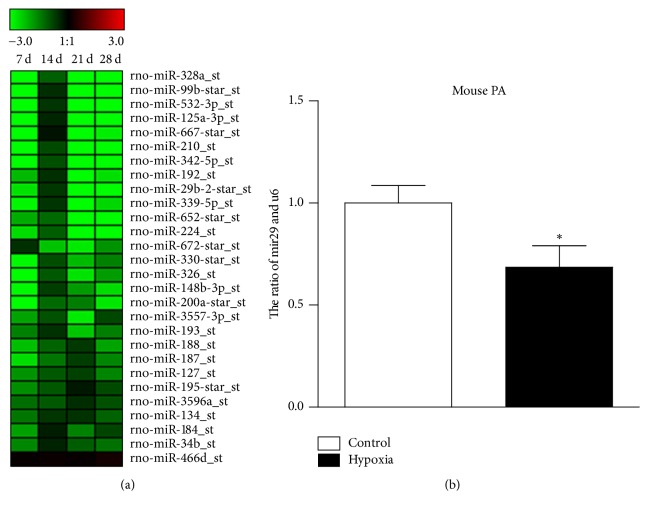
*The expression of miR-29b is downregulated in PAH*. (a) A cluster analysis of the miRNA expression from individual specimens was assessed by a microarray analysis (*n* = 6 rats per time point). (b) The level of miR-29b expression in the pulmonary artery tissue of mice exposed to hypoxia or normal air (control group) (*n* = 6 mice/group). ^*∗*^*p* < 0.05.

To determine the lung miRNA profile in PAH mice model, we performed a microarray analysis. The microarray profile revealed that several miRNAs, including miR-328a, miR-99b, miR-210, miR-342, miR-29b, miR-224, and miR-339, were regulated at different time points. Among them, the expression of miR-29b was significantly decreased after three weeks (Figure 1(a)). A quantitative real-time polymerase chain reaction (qRT-PCR) assay was performed to confirm the expression of miR-29b in the pulmonary artery tissue in hypoxia PHA mice (Figure 1(b)).

### 3.2. MiR-29b Affects the Proliferation and Apoptosis of PASMCs

**Figure 2:**
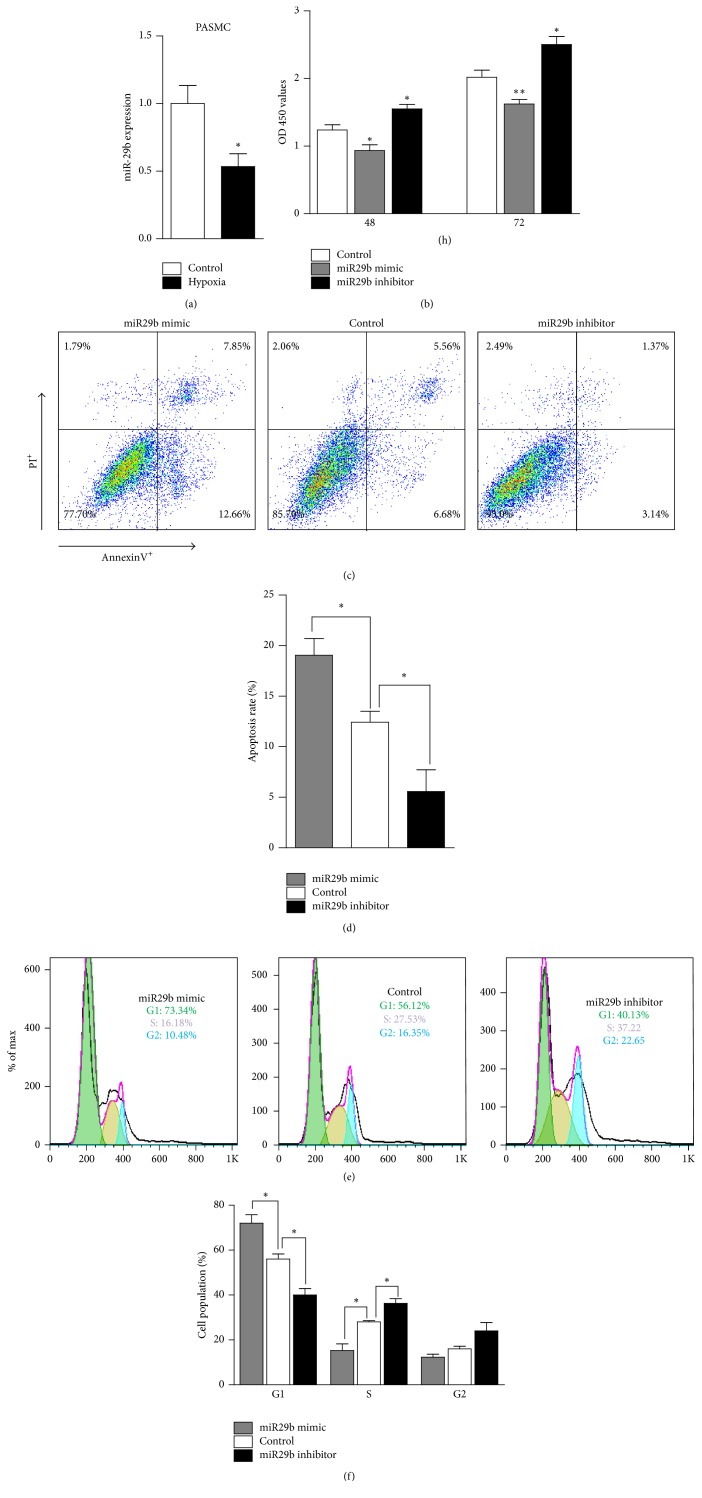
*MiR-29b promotes apoptosis of PASMCs*. (a) The expression of miR-29b in PASMCs cultured under hypoxia or normal air (control) for 48 h (*n* = 6 mice/group). (b) The proliferation of PASMCs following transfection with the miR-29b mimic, inhibitor, or negative control for 48 h or 72 h analyzed using a CCK-8 assay kit. The OD450 value revealed the number of PASMCs (*n* = 6 mice/group). (c and d) The representative graph and statistical chart of the apoptosis rate of PASMCs treated as indicated for 48 h determined by flow cytometry following AnnexinV and PI staining. (e and f) The representative graph and statistical chart of the number of PASMCs treated with the miR-29b mimic, inhibitor, or negative control for 48 h in the G0/G1, S, and G2/M phases determined by flow cytometry after staining with PI. ^*∗*^*p* < 0.05 and ^*∗∗*^*p* < 0.01.

PASMCs play a significant role in the progression PAH. To access the role of miR-29b in modulating biological functions in PAH, we used PASMCS to do the following study. First, we found that the expression of miR-29b was significantly decreased in PASMCS after 48 h of hypoxia exposure (Figure 2(a)). Then PASMCs were transfected with a miR-29b mimic or inhibitor, and the effect of miR-29b on the proliferation of PASMCs was examined 48 h and 72 h following the transfection. We used a CCK8 kit for processing and then measured the OD (optical density) values. All OD values were minus the blank control group (no cell) of OD. Result revealed that overexpression of miR-29b significantly decreased the OD of the PASMCs compared to the negative controls. In contrast, the OD of the PASMCs transfected with the miR-29b inhibitor was significantly increased compared to the negative control-transfected cells (Figure 2(b)). Next, we used an AnnexinV-FITC/PI dual staining method for detecting apoptosis of the PASMCs. Flow cytometry indicated that the early apoptosis rate of the PASMCs was 7.85%, and the late apoptosis rate was 12.66%, a total of 8.27% higher than that of the control (5.56% for early, 6.68% for late, and 12.24% total apoptosis) (Figures 2(c) and 2(d)). The total apoptosis percentage of the PASMCs transfected with the miR-29b inhibitor was 4.51%, 7.73% lower than that of the control (Figures 2(c) and 2(d)). These data indicate that miR-29b inhibits PASMC proliferation by inducing cell cycle arrest at the G1/S phase and increases apoptosis.

The imbalance between proliferation and apoptosis may lead to changes in the number of PASMCs. To determine whether the altered cellular proliferation was a result of the cell cycle, flow cytometric analysis was conducted. After transfecting the cells for 48 h, flow cytometry revealed that the percentage of miR-29b mimics in the G1 phase was 73.34%. This value was 17.22% higher than that of control (56.12% in the S phase). The percentage of PASMCs transfected with the miR-29b inhibitor in the G1 phase was 40.13%, a value 15.99% lower than that of the controls (Figures 2(e) and 2(f)). These results indicate the miR-29b mimic increased the proportion of PASMCs in the G1 phase and decreased the proportion of PASMCs in the S phase compared with the cells transfected with the control.

### 3.3. MiR-29b Targets 3′ UTR of Mcl-1 and CCND2 and Inhibits the Expression of Mcl-1 and CCND2

**Figure 3:**
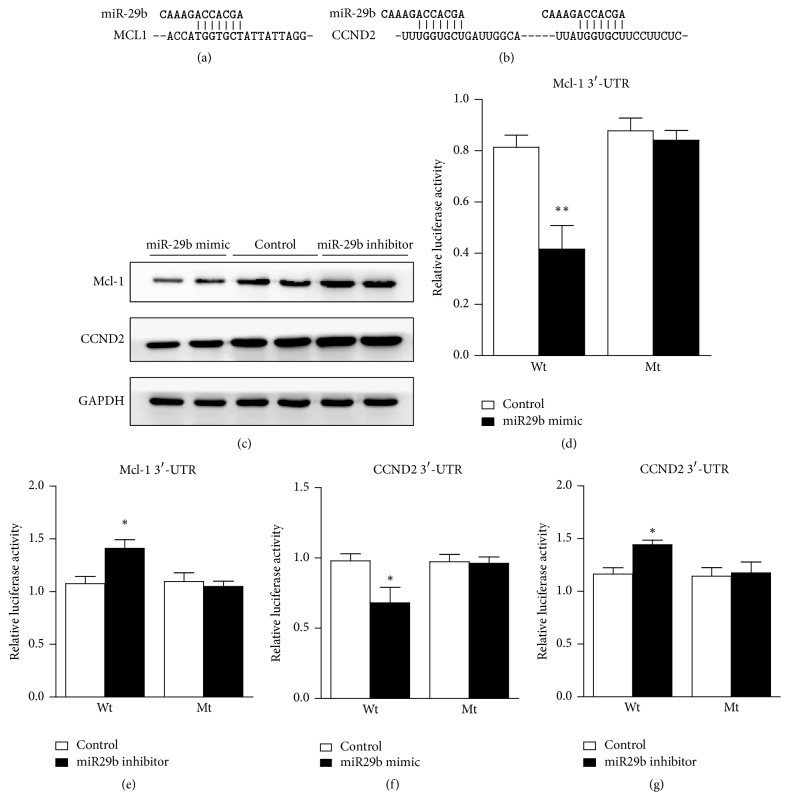
*MiR-29b downregulates the expression of Mcl-1 and CCND2*. (a and b) The potential binding sites of miR-29b on the 3′ UTR of Mcl-1 and CCND2. (c) The expression of Mcl-1 and CCND2 in PASMCs treated with the miR-29b mimic, inhibitor, or control by Western blot. (d and e) The effect of the miR-29b mimic and inhibitor on the wildtype and mutant 3′ UTR of Mcl-1 as analyzed by a luciferase assay. (f and g) The effect of the miR-29b mimic and inhibitor on the wildtype and mutant 3′ UTR of CCND2 as analyzed by a luciferase assay. ^*∗*^*p* < 0.05 and ^*∗∗*^*p* < 0.01.

The ability of miR-29b to impede PASMC proliferation and apoptosis may be due to its ability to pleiotropically regulate genes in diverse aspects of these processes. To identify the effectors of miR-29b, a bioinformatic analysis was performed to search for potential regulatory targets of miR-29b. Figures 3(a) and 3(b) show the sequences of the 3′UTRs of Mcl-1 and CCND2 that represent the binding sites of miR-29b. To examine whether miR-29b alters the expression of Mcl-1 and CCND2, we performed a Western blot analysis of the PASMCs transfected with the miR-29b mimic, inhibitor, or control. Our results demonstrated a significant downregulation of Mcl-1 and CCND2 in the PASMCs transfected with the miR-29b mimic (Figure 3(c)). Further we did the luciferase assay to detect if miR-29b targets Mcl1 or CCDN2. Results revealed that miR-29b targets 3′ UTR of Mcl-1 (Figures 3(d) and 3(e)) and CCND2 (Figures 3(f) and 3(g)).

**Figure 4:**
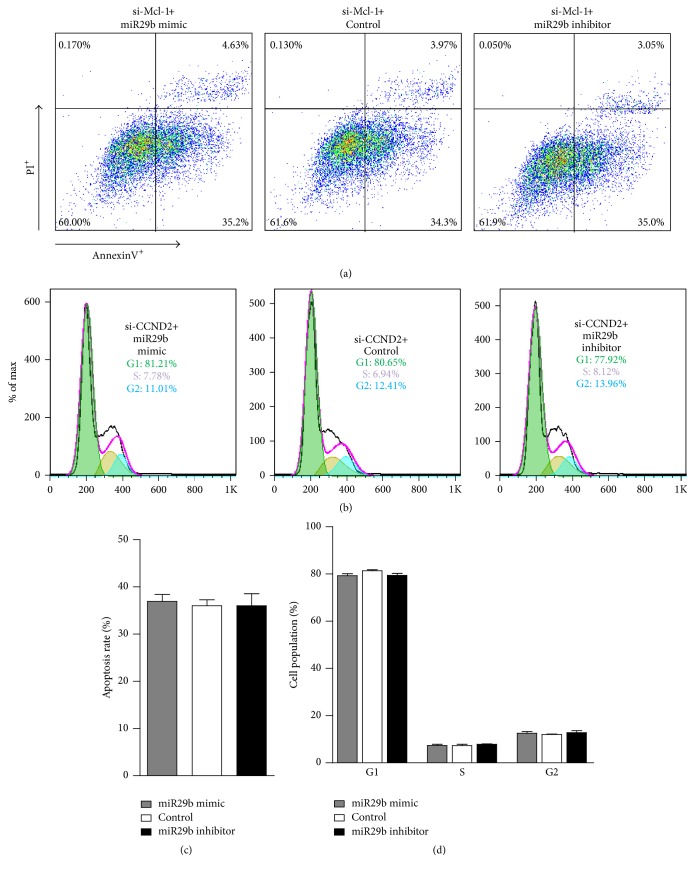
*Mcl-1 and CCND2 silencing results in increased PASMC apoptosis and rescues the PASMC G1/S delay, respectively, in response to miR-29b expression*. (a and c) The representative graph and statistical chart of the PASMC apoptosis rate for each indicated treatment for 48 h after si-Mcl-1 transfection as determined by flow cytometry following AnnexinV and PI staining. (b and d) Representative dot plots and statistical chart of the number of PASMCs treated with the miR-29b mimic, inhibitor, or negative control for 48 h after si-CCND2 transfection in the G0/G1, S, and G2/M phases.

To determine if miR-29b regulates the proliferation and apoptosis of PASMCs through Mcl-1 and CCND2, we used siRNA interference against Mcl-1 and CCND2. After silencing Mcl-1, the percentage of apoptosis between the miR-29b mimic, miR-29b inhibitor, and control was also very similar (Figures 4(a) and 4(c)). Furthermore, after silencing CCND2 expression, the percentage of PASMCs transfected with the miR-29b mimic was 81.21% and 7.78% in the G1 and S phase, respectively (Figures 4(b) and 4(d)). These values were close to the percentage exhibited by the controls, which were 80.65% and 6.94%, respectively. And the percentages were the same as that observed between the miR-29b inhibitor and control. We did not find any significant differences between the three groups. Together, these results suggest that miR-29b suppresses proliferation and promotes apoptosis in PASMCs by inhibiting the expression of Mcl-1 and CCND2.

## 4. Discussion

MiRNAs are a class of small (19–25 nucleotides) noncoding and highly conserved single-stranded RNAs that repress protein translation through binding to the 3′ UTR of their target mRNAs in a sequence-specific manner. They are particularly important for the regulation of cellular proliferation, cycle, differentiation, survival, and apoptosis [18]. Recently, several studies have found that miRNAs play an important role in cardiac development, myocardial hypertrophy, atherosclerosis, heart failure, arrhythmia, and other cardiovascular diseases [19]. MiR-34a has been shown to promote the proliferation of human PASMCs by targeting PDGFRA [20]. Moreover, miR-103/107 is also involved in the hypoxia-induced proliferation of PASMCs by targeting HIF-1*β* [21]. In addition, miRNA-223 has been found to attenuate hypoxia-induced vascular remodeling by targeting RhoB/MLC2 in PASMCs [22]. However, studies regarding miR-29b in the pathogenesis of PAH are limited. In this study, we have demonstrated that (1) miR-29b expression is downregulated in animal models of PAH induced by hypoxia and (2) Mcl-1 and CCND2 are the target genes of miR-29b. Furthermore, miR-29b inhibits the proliferation and promotes the apoptosis of PASMCs by suppressing the expression of Mcl-1 and CCND2.

MiR-29b is downregulated in a variety of diseases. Cushing et al. found that the miR-29 family is suppressed by transforming growth factor- (TGF-) *β*1 in human fetal lung fibroblast IMR-90 cells, and many fibrosis-associated genes upregulated by TGF-*β*1 are depressed by knocking down miR-29b [10]. An analysis of the miRNA expression profiles and target genes in Ewing's Sarcoma revealed that 58 out of the 954 analyzed miRNAs were significantly differentially expressed, including miR-29b [23]. In addition, the expression of miR-29 in several types of cancer also appears to be downregulated. Therefore, miR-29b is often used as a tumor suppressor factor, particularly in cancer research [11, 12, 15]. Since miR-29b directly targets collagen mRNA, it was found to be consistently reduced in both partial nephrectomy and marinobufagenin-infused animals. In our study, the gene chip showed that miR-29b expression was downregulated in our PAH mice model. Furthermore, miR-29b expression was significantly decreased in the pulmonary artery tissues of mice exposed to hypoxia, respectively. These results are consistent with previous reports.

The pathogenesis of miR-29b has remained largely unknown. The bioinformatic algorithms of the present study predicted that Mcl-1 and CCND2 were the target genes of miR-29b. CCND2 forms a complex with CDK4 or CDK6 and functions as a regulatory subunit, leading to the phosphorylation of pRb, and the release of the transcription factor E2F from pRb-mediated inhibition. E2F can activate thymidylate, thymidine kinase, or DNA polymerase and participates in DNA synthesis as well as gene transcription, finally initiating cells to enter into S phase. The accumulation of CCND 2 can isolate the cell cycle inhibitor, P27, and play a critical role in the progression of cells from G1 into S phase. Previous studies have focused on CCND2 for the regulation of smooth cells in tumors [24]. For example, researchers found that the introduction of miR-29b in Ewing's Sarcoma cells could inhibit the c-Myc-mediated upregulation of CCND2, which resulted in the prevention of cell cycle progression [15]. In addition, CCND2 can be regulated by miR-206 [25], miR-340 [26], miR-154 [27], miR-133a [28], miR-155 [29], miR-1 [30], miR-16 [31], miR-610 [32], miR-26a [33], miR-195 [34], and miR-204 [35], which have roles in cellular proliferation disorders (e.g., breast cancer, cervical carcinoma, and retinoblastoma). Here, we performed* in vitro* and* in vivo* studies to demonstrate that miR-29b can regulate PASMC proliferation and G1/S transition through targeting CCND.

Due to internal and external environmental changes, death signals are triggered and cause cell death known as apoptosis. At present, there are three main pathways of cellular apoptosis: (1) the endogenous pathway (mitochondrial apoptosis pathways); (2) the exogenous pathway (death receptor apoptosis pathway); and (3) the endoplasmic reticulum apoptosis pathway.

The loss of apoptosis signaling leads to the occurrence of many diseases. Moreover a decrease in PASMC apoptosis in the PAH pathological process breaks the stability of the internal environment. In this study, we focused on whether miR-29b is capable of reducing PASMC proliferation and promoting apoptosis. Mcl-1 is an antiapoptotic Bcl-2 family member that interacts with members of the BH3 family (e.g., NOXA, Bim, Bid, and Puma), decreases mitochondrial membrane permeability, blocks the activity of Bax and Bak, and ultimately inhibits apoptosis. We infer that miR-29b downregulates Mcl-1, thereby promoting apoptosis. Previous studies found that miR-29b can directly downregulate the translation of Mcl-1, thus inhibiting prostate cancer cells and extrahepatic bile duct cancer cell line tumor progression [16, 17]. A significantly lower expression of miR-29b and a higher expression of the potential target gene Mcl-1 were found in peripheral blood mononuclear cells from acute and chronic myeloid leukemia patients compared with a group of healthy individuals [36]. Moreover, Toll-like receptor (TLR) pathway-activated plasmacytoid dendritic cells (pDCs) are resistant to glucocorticoid- (GC-) induced apoptosis. Hong et al. [19] found that miR-29b was involved in TLR-inhibited GC-induced pDC apoptosis by directly targeting Mcl-1. This supports the theory that miR-29b is a proapoptotic factor. However, in a study of spinal cord injury [37], miR-29b was shown to play a repressive role on apoptotic BH3-only genes including Puma, NOXA, Bid, and Bad, but not Mcl-1. The different roles exhibited by miR-29b regarding Mcl-1 expression between this previous study and our report (including previous work by other researchers) indicates a cell-specific regulation of miR-29b regarding apoptosis.

Previous studies have indicated that miR-29b has a therapeutic effect for many cardiovascular diseases. Merk et al. [38] showed that miR-29b expression was increased in the Marfan ascending aorta during early aneurysm development, and miR-29b oligonucleotide inhibitors prevented early aneurysm formation in Marfan mice. It is also found that miR-29b has an antifibrotic effect on the development of pulmonary fibrosis [39]. A miRNA can regulate multiple target genes, and multiple miRNAs can regulate a protein-coding gene. Therefore, miR-29b may have several target genes in PAH and could be involved in a wide range of cell signaling pathways through these target genes. Many researchers have reported that target genes of miR-29b could activate JNK-GATA3, TGF-*β*, MAPK, and other signal transduction pathways [40–42]. In particular, the TGF-*β* pathway is increasingly recognized as a signal transduction pathway involved in cellular proliferation, differentiation, apoptosis, and immune regulation, among other important biological processes. In our PAH mice model, miR-328, miR-210, miR-342, and miR-125 were also downregulated in addition to miR-29b. Therefore, we wonder whether there is a close relationship between these miRNAs and the intersection between their target genes. If such a link does exist, whether there is a balance in their roles for regulating PAH should be addressed. These questions are worthy of future study.

The limitations of the present study include that we did not analyze the correlation between PAH cases and miR-29b in clinical samples. If possible, further study of miR-29b changes in the peripheral blood of children with PAH may provide insight into the potential use of miR-29b for diagnosis and as a biomarker of PAH. In addition, the upstream gene regulation of miR-29b has not been studied. Future studies should focus on the upstream regulation points which influence the expression of miR-29b. At that time, we can find a specifically regulated pathway with miR-29b as the center.

Taken together, our work has demonstrated that miR-29b is downregulated in PAH. Reintroducing miR-29b expression was capable of inhibiting CCND and Mcl-1, which suppressed cellular proliferation and induced apoptosis in PASMCs, respectively. These findings strongly indicate that miR-29b may function as a novel therapeutic target for patients with PAH.
